# The Importance of Instrumentation Length in Ankylosing Spinal Disorders and Thoracolumbar Fractures

**DOI:** 10.3390/jcm15135082

**Published:** 2026-06-30

**Authors:** Federico Fusini, Alessandro Rava, Giosuè Gargiulo, Domenico Messina, Alberto Lorenzi, Silvia Amico, Gabriele Colò, Massimo Girardo

**Affiliations:** 1 Spine Surgery Unit, Orthopaedic and Trauma Centre, Città della Salute e della Scienza di Torino, 10126 Turin, Italy; gargiulogiosue2@gmail.com (G.G.); dr.domenicomessina@gmail.com (D.M.); allorenzi@cittadellasalute.to.it (A.L.); samico@cittadellasalute.to.it (S.A.); massimogirardo.cto@gmail.com (M.G.); 2 Circolo Hospital, Macchi Foundation, Insubria University, Viale Borri 57, 21100 Varese, Italy; gabriele.colo@yahoo.it

**Keywords:** fracture, ankylosing, spondylitis, spine, trauma, posterior instrumentation, ankylosing spinal disorders, DISH, thoracolumbar, pedicle screw

## Abstract

**Background/Objectives**: Ankylosing Spinal Disorders (ASDs) encompass a heterogeneous group of rheumatic diseases characterized by progressive ankylosis of the axial skeleton, including Ankylosing Spondylitis (AS), Diffuse Idiopathic Skeletal Hyperostosis (DISH), and Non-Radiographic Axial Spondyloarthritis (nr-AxSpA). Spinal ankylosis profoundly alters the biomechanical properties of the vertebral column, transforming it into a rigid long-bone equivalent and dramatically increasing fracture risk even after low-energy trauma. Once a fracture occurs, the long lever arm created by the ankylosed segments generates enormous mechanical stress at the fracture site, making surgical stabilization mandatory in the vast majority of cases. Long posterior instrumentation is the treatment of choice; however, no consensus exists regarding the optimal number of instrumented levels. The aim of this study is to clinically and radiologically evaluate long posterior instrumentation in the 3 + 3 (3 proximal and 3 caudal screws), 3 + 2 (3 proximal and 2 caudal screws), or 2 + 2 (2 proximal and 2 caudal screws) configuration for the treatment of traumatic ASD thoracolumbar vertebral fractures, in terms of implant failure, infection rate, and mortality. **Methods**: Between 2018 and 2023, 65 consecutive patients with ASD-related thoracolumbar vertebral fractures were treated at our institution. After applying pre-defined inclusion and exclusion criteria, 37 patients were enrolled. Patients were retrospectively divided into three groups according to the posterior arthrodesis configuration (notation indicates number of instrumented vertebral levels proximal + distal to the fracture: 3 + 3, 3 + 2, or 2 + 2). Radiological outcomes were assessed for loosening, screw cut-out, and implant breakage. Infection and mortality rates within 3 months from surgery were evaluated as secondary endpoints. Statistical analysis was performed using the Fisher exact test (significance set at *p* < 0.05). **Results**: Thirty-seven patients (28 males and 9 females; mean age 77 ± 7.3 years) were included, with a mean follow-up of 30 ± 5.3 months. Instrumentation configurations were as follows: 23 (3 + 3), 5 (3 + 2), and 9 (2 + 2). Three implant failures (8.1%) and four infections (10.8%) were recorded. Eleven patients died within 3 months of surgery. A statistically significant difference was found between instrumentation length and mechanical complications (*p* = 0.0468), while no significant difference was observed for infection (*p* = 1) or mortality rate (*p* = 0.137). **Conclusions**: In this exploratory retrospective cohort, the 3 + 3 configuration was associated with the lowest observed rate of implant failure in ASD thoracolumbar fractures, suggesting a potential mechanical advantage over shorter constructs that warrants confirmation in larger prospective studies. No significant correlation was found between instrumentation length and infection rate or early mortality. Prospective, multicentre studies with larger cohorts are warranted to establish definitive guidelines for instrumentation length in this challenging patient population.

## 1. Introduction

Ankylosing Spinal Disorders (ASDs) represent a heterogeneous group of rheumatic conditions characterized by progressive ossification and ankylosis of the axial skeleton. The most common forms include Ankylosing Spondylitis (AS), Diffuse Idiopathic Skeletal Hyperostosis (DISH), and Non-Radiographic Axial Spondyloarthritis (nr-AxSpA) [1]. AS is a chronic immune-mediated inflammatory arthropathy predominantly affecting the sacroiliac joints and spine, leading to the formation of marginal syndesmophytes, progressive kyphosis, and ultimately a bamboo spine radiographic appearance. DISH, in contrast, is a non-inflammatory degenerative condition characterized by flowing ossification of the anterolateral vertebral ligaments and enthesopathies, affecting predominantly the thoracic spine of elderly individuals. Both conditions share a fundamental biomechanical consequence: the vertebral column is transformed from a segmented, shock-absorbing structure into a rigid, lever-like construct that concentrates mechanical stress at any discontinuity, including fracture sites [2].

Spinal deformities and rigidity in ASD patients lead to increased fragility of the vertebral column and severely restricted range of motion [2]. The combination of syndesmophyte formation, progressive kyphosis, and the frequent co-existence of osteoporosis—often masked by falsely elevated dual-energy X-ray absorptiometry (DEXA) scores—makes the ASD spine highly susceptible to fractures, even following apparently trivial low-energy trauma such as minor falls or simple mechanical stress [3]. The risk of vertebral fractures in ASD patients is estimated to be between 10% and 14%, significantly exceeding that of age-matched controls [3,4]. The lower cervical spine represents the most frequently affected region (approximately 78% of fractures), followed by the thoracolumbar junction (approximately 20%) [4]. In approximately 8% of cases, fractures involve multiple non-contiguous levels—a phenomenon known as polyfocal fracture—carrying an even worse neurological prognosis [4].

The neurological consequences of spinal fractures in ASD are particularly severe. The risk of nerve root or spinal cord injury, intramedullary oedema, and spinal epidural hematoma is more than 11 times higher in ASD patients than in the general population [5], with spinal cord injury carrying a particularly unfavorable prognosis in this fragile population [6]. Acute neurological complications are reported in 23% to 33% of cases depending on the spinal level, and secondary neurological deterioration—arising from progressive instability at the fracture site—may occur in up to 15% of initially neurologically intact patients [7]. These data underscore the critical importance of achieving immediate and robust mechanical stabilization at the time of surgery.

Despite broad surgical consensus in favor of long posterior instrumentation for thoracolumbar ASD fractures, the optimal number of instrumented levels above and below the fracture remains a matter of debate. The German Society for Orthopaedics and Trauma (DGOU) currently recommends a minimum of two pedicle fixation levels proximal and distal to the fracture [8]. However, this recommendation is largely based on expert opinion and small retrospective series, and no high-level evidence has been published to definitively resolve the question of whether 2 + 2, 3 + 2, or 3 + 3 configurations offer meaningfully different mechanical outcomes [9]. Additionally, the role of cement augmentation with polymethylmethacrylate (PMMA) in improving screw purchase in the context of ASD-related osteoporosis remains incompletely characterized.

Against this background, the aim of the present study is to clinically and radiologically evaluate long posterior instrumentation in the 3 + 3, 3 + 2, and 2 + 2 configurations for the treatment of traumatic ASD thoracolumbar vertebral fractures at a single tertiary spine referral center, with a focus on implant failure rates, infection, and early mortality.

## 2. Materials and Methods

### 2.1. Study Design and Patient Selection

This is a single-center, retrospective, observational comparative study. Between January 2018 and December 2023, 65 consecutive patients with thoracolumbar vertebral fractures in the context of ASD were treated by the same dedicated spine surgery team at the Spine Surgery Unit, Orthopaedic and Trauma Centre, Città della Salute e della Scienza di Torino, Turin, Italy. This retrospective observational study, based on anonymized pre-existing data, was conducted in accordance with the Declaration of Helsinki (1964 and further amendments) and was exempt from mandatory Ethics Committee approval under Italian national legislation (D.Lgs. 211/2003); formal IRB approval was therefore not required. Written informed consent was obtained from all patients or, in the case of deceased patients, from their nearest legal representative.

ASD pathologies included in the study were Ankylosing Spondylitis, Diffuse Idiopathic Skeletal Hyperostosis (DISH, defined according to Resnick’s radiological criteria), and Non-Radiographic Axial Spondyloarthritis [8]. No restriction was applied based on patient age or sex.

### 2.2. Inclusion and Exclusion Criteria

Patients were included if they presented with thoracolumbar fractures (i.e., between T10 and L2) classified as AOSpine Thoracolumbar Spine Injury Classification System [10] types B or C, at a single level, with or without neurological impairment. Cervical fractures, thoracocervical junction fractures (above T3), and fractures at other levels were excluded. Patients with spondylodiscitis-related fractures, Anderson lesions, and multiple-level (polyfocal) fractures were also excluded, as these represent distinct pathological and biomechanical entities requiring different surgical strategies.

Following application of inclusion and exclusion criteria, 37 patients were enrolled in the final analysis and retrospectively divided into three groups based on the posterior arthrodesis configuration employed: Group A (3 + 3 configuration, *n* = 23), Group B (3 + 2 configuration, *n* = 5), and Group C (2 + 2 configuration, *n* = 9).

### 2.3. Surgical Technique

All patients underwent open posterior spinal instrumentation using a standard midline approach. The choice of instrumentation length (3 + 3, 3 + 2, or 2 + 2) was determined intraoperatively based on the individual surgeon’s assessment of bone quality, fracture pattern, and patient physiological reserve, in the absence of pre-defined a priori criteria—a limitation acknowledged and discussed later in this manuscript. No intraoperative navigation or robotic assistance was employed. Instrumentation was performed using standard polyaxial pedicle screw-rod constructs [11].

In a subset of patients with clinically or radiologically suspected poor bone quality (*n* = 3, 8.1%), PMMA cement augmentation was performed through the inner core of fenestrated cannulated screws. Cement volumes of 2 cc and 3 cc were injected for thoracic and lumbar screws, respectively, after confirming adequate screw positioning under fluoroscopic guidance and ensuring no cement leakage into the spinal canal. No anterior stabilization was performed in any patient.

### 2.4. Postoperative Management and Follow-Up

All patients underwent standard postoperative mobilization protocols, with early rehabilitation initiated within 48 to 72 h of surgery when clinical conditions permitted, consistent with previously reported postoperative mobilization strategies in ASD patients [12]. Plain radiographs (anteroposterior and lateral projections) were obtained the day after surgery and at 1, 3, 6, and 12 months postoperatively, and then annually thereafter. Computed tomography (CT) scanning was performed when implant failure or pseudoarthrosis was clinically or radiologically suspected.

Radiological assessment focused on: (1) number of instrumented segments; (2) evidence of screw loosening, defined as a peri-screw radiolucency ≥ 1 mm on plain radiographs; (3) screw cut-out; and (4) rod or implant breakage. All radiological assessments were performed by two independent senior spine surgeons blinded to group allocation; discrepancies were resolved by consensus. The infection rate (superficial and deep) and mortality rate were evaluated as secondary endpoints at 3 months from the index surgical procedure.

### 2.5. Statistical Analysis

Continuous variables were expressed as mean ± standard deviation (SD). Categorical variables were reported as absolute numbers and percentages. Inter-group comparisons for categorical outcomes (mechanical complications, infection, and mortality) were performed using the Fisher exact test, selected given the small sample sizes and non-normal distribution of categorical variables. Modal distribution was used to identify the preferred configuration across the cohort. The level of statistical significance was set at *p* < 0.05. All statistical analyses were performed using standard statistical software (IBM SPSS Statistics, version 28.0; IBM Corp., Armonk, NY, USA).

## 3. Results

### 3.1. Demographic and Clinical Characteristics

After applying inclusion and exclusion criteria, 37 patients (28 males and 9 females; mean age 77 ± 7.3 years; range 58–91 years) were enrolled in the study. The underlying ASD diagnosis was Ankylosing Spondylitis in the majority of cases, with a subset affected by DISH or nr-AxSpA. According to the AOSpine classification, fractures were type B in 27 patients (11 B1, 7 B2, 10 B3) and type C in 10 patients. Neurological impairment was documented at admission in 7 of 37 patients (18.9%). The mean follow-up duration was 30 ± 5.3 months. Eleven patients (29.7%) died within 3 months of surgery. Main demographic data and fracture characteristics are summarized in Table 1.

### 3.2. Instrumentation and Surgical Data

**Figure 1:**
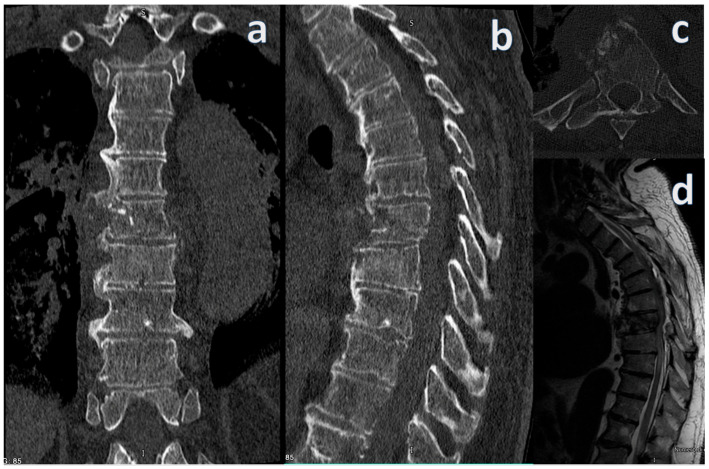
70-year-old male patient affected by D7 fracture (AO type B) and spinal cord injury ASD. (**a**) CT scan coronal view (**b**) CT scan lateral view (**c**) CT scan axial view (**d**) MRI lateral view.

**Figure 2:**
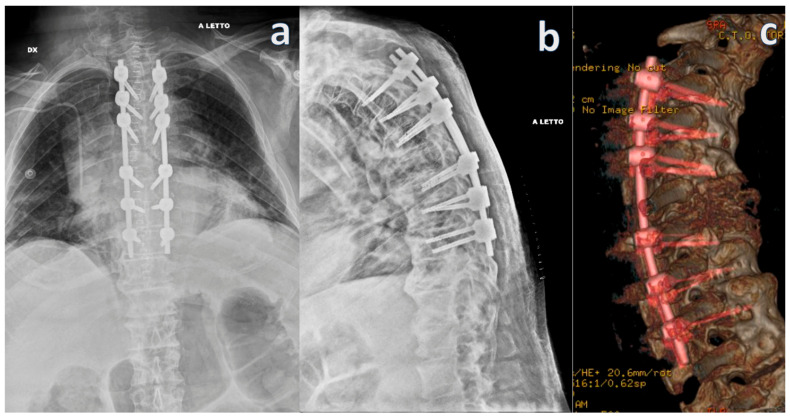
Surgical 3 + 3 stabilisation and decompression treatment in 70-year-old male patient affected by D7 fracture (AO type B) and spinal cord injury in ASD. (**a**) after surgery X-ray frontal view (**b**) after surgery X-ray lateral view (**c**) 3D CT scan.

The mean number of instrumented segments was 5.3 ± 0.5 (range 4–6). Modal distribution analysis confirmed a strong institutional preference for the 3 + 3 configuration (23/37, 62.2%), followed by the 2 + 2 (9/37, 24.3%) and 3 + 2 (5/37, 13.5%) configurations. Mean screw density was 0.89 ± 0.06, reflecting the decision to instrument the fractured vertebra in a subset of patients when biomechanical or fracture pattern considerations warranted it. Cement augmentation was employed in only 3 patients (8.1%), precluding any meaningful statistical comparison between cemented and uncemented constructs within the current cohort. A clinical case of a 70-year-old male patient affected by D7 fracture (AO type B) and spinal cord injury in ASD is presented in Figure 1 and Figure 2. The patient underwent urgent 3 + 3 stabilization and decompression treatment.

### 3.3. Mechanical Complications

Three implant failures were recorded overall (8.1%), all requiring surgical revision: two cases of screw loosening (both in the 2 + 2 group, representing 22.2% of that group) and one case of rod rupture (in the 3 + 3 group, 4.35% of that group), the latter associated with pseudoarthrosis at the fracture site. No implant failures were observed in the 3 + 2 group. Statistical analysis demonstrated a significant difference in mechanical complication rates between instrumentation configurations (*p* = 0.0468), with the 2 + 2 group exhibiting the highest rate. These findings suggest a possible association between instrumentation length and construct mechanical durability in the context of ASD, which warrants confirmation in larger, prospective studies.

### 3.4. Infectious Complications

Four infections were recorded during the follow-up period (10.8% overall): three superficial and one deep. Superficial infections occurred in 2 patients with a 3 + 3 construct (8.7%) and 1 patient with a 2 + 2 construct (11.1%). The single deep infection was identified in the 3 + 2 group (20%). No statistically significant difference was found between instrumentation length and infection rate (*p* = 1). The overall infection rate is consistent with published literature reporting rates of 4.2% to 17.2% in ASD patients undergoing posterior spinal surgery, significantly higher than in the general surgical population, largely attributable to the immune dysregulation inherent to inflammatory rheumatic diseases [13,14,15,16].

### 3.5. Mortality

The 3-month mortality rate was 29.7% (11/37 patients). Pulmonary complications were responsible for approximately 70% of deaths, occurring predominantly in patients with multiple cardiorespiratory comorbidities. A subset of these patients had also received cement augmentation; however, given the very small number of cemented cases (*n* = 3), no causal or statistically meaningful relationship between PMMA augmentation and pulmonary complications can be inferred from the present data. The remaining deaths were attributable to systemic causes unrelated to the surgical procedure. No statistically significant association was found between instrumentation configuration and 3-month mortality (*p* = 0.137). The high overall mortality rate, compared to the 15% reported by Ull et al. [16], is largely explained by the advanced mean age of our cohort (77 years), the high burden of systemic comorbidities, and the definition of the endpoint at 3 months postoperatively—a period encompassing the entire in-hospital stay (mean 55.7 ± 60.9 days) during which 52% of patients were lost to outpatient follow-up. These findings are consistent with the data reported by Chen et al. [17] and Sharma et al. [18], who identified advanced age and systemic comorbidities as independent predictors of early mortality in ASD fracture patients.

## 4. Discussion

The management of thoracolumbar fractures in patients with ASD remains one of the most challenging problems in spinal surgery. The peculiar biomechanical environment created by spinal ankylosis—with its rigid, lever-like construct, the omnidirectional instability of AO type B and C fractures, and the frequent coexistence of subclinical osteoporosis—demands a surgical strategy that is simultaneously robust, durable, and tolerated by a frail, elderly patient population [9,19].

The fundamental biomechanical rationale for extended posterior instrumentation in ASD fractures lies in the concept of stress distribution. The ankylosed spine behaves as a long bone, and any fracture across this rigid structure is subjected to enormous bending, shear, and torsional forces at the fracture site. Biomechanical studies have demonstrated that the bamboo spine creates a lever arm of exceptional length that concentrates mechanical stress far beyond what short-segment constructs can withstand [20]. In this context, extending pedicle screw fixation to three levels above and below the fracture distributes these forces over a greater number of anchor points, reducing the load per screw and diminishing the risk of loosening or pull-out—particularly critical in bone of compromised quality [8,19].

The present study provides direct clinical evidence supporting this biomechanical reasoning. The 2 + 2 configuration was associated with a mechanical failure rate of 22.2% within its group, compared to 4.35% in the 3 + 3 group—a statistically significant difference (*p* = 0.0468). Importantly, the single failure in the 3 + 3 group was attributable to pseudoarthrosis at the fracture site rather than to a biomechanically insufficient construct per se. These results are clinically meaningful: all three implant failures required surgical revision, exposing already frail and elderly patients to the significant additional risks of reoperation. This finding is in partial contrast with Svac et al. [21], who reported no significant difference between long and short-segment stabilisation; however, a critical methodological distinction must be noted. In their series, some vertebrae were intentionally skipped within long-segment constructs, resulting in a non-contiguous fixation pattern. In our study, pedicle fixation density was maintained at 100%—i.e., every vertebral level within the instrumented segment received bilateral pedicle screws—yielding superior mechanical continuity and load distribution. Our findings are broadly consistent with those of Sulpis et al. [22], who similarly reported favorable clinical and radiological outcomes with isolated posterior stabilisation in type B and C thoracolumbar ASD fractures, and align with the management principles outlined in recent reviews on the surgical treatment of thoracolumbar fractures and dislocations in ankylosing spinal conditions [23].

The role of bone mineral density in ASD fracture surgery deserves specific consideration. As highlighted by Klingberg et al. [14] and Hasegawa et al. [24], bone mineral density in ASD patients is frequently underestimated by standard DEXA measurements due to syndesmophyte-induced artefactual elevation of apparent bone density scores. Subclinical osteoporosis is thus highly prevalent in this population, significantly compromising screw purchase and increasing the risk of hardware loosening or pull-out. PMMA cement augmentation through fenestrated cannulated screws has been demonstrated to increase pull-out strength in osteoporotic bone to values exceeding those of normal bone [13,25], and represents a potentially valuable adjunct in patients with low Hounsfield Unit values on preoperative CT scanning. In our series, the small number of cemented cases (*n* = 3) precluded formal statistical comparison between cemented and uncemented constructs. Future studies should specifically address the interaction between instrumentation length, cement augmentation, and bone quality—particularly in relation to the Hounsfield Unit threshold below which augmentation should be systematically employed.

With respect to infectious complications, the overall rate of 10.8% in our cohort falls within the range of 4.2% to 17.2% reported in the contemporary ASD surgical literature [13,15,16]. The immune dysregulation intrinsic to inflammatory rheumatic diseases—particularly the T cell-mediated pathways central to AS pathogenesis—increases susceptibility to both superficial and deep surgical site infections [14]. Notably, the single deep infection in our series occurred in the 3 + 2 configuration group, and no statistically significant difference was found across groups (*p* = 1). This suggests that infection risk in ASD surgery is predominantly determined by patient-related factors (immunosuppressive treatment, nutritional status, comorbidities) rather than by the specific instrumentation construct employed.

The 3-month mortality of 29.7% observed in this cohort is high by any standard and reflects the extraordinary vulnerability of this patient population. The advanced mean age (77 years), the high prevalence of cardiorespiratory comorbidities, and the prolonged in-hospital course (mean stay 55.7 ± 60.9 days) collectively contribute to this outcome. Pulmonary complications—including pneumonia, pulmonary embolism, and respiratory failure—were responsible for the majority of deaths, consistent with the recognized propensity for thoracic cage rigidity in ASD to compromise respiratory mechanics, particularly in the perioperative period. These findings reinforce the importance of early and aggressive respiratory physiotherapy, careful anesthetic planning, and systematic multidisciplinary perioperative management in this population [18].

### Limitations

This study has several limitations that warrant consideration. First, its retrospective, single-center design and the relatively small and unevenly distributed sample size across the three instrumentation groups (notably the small 3 + 2 group, *n* = 5) limit the statistical power of the analysis and the generalizability of the findings; the borderline *p*-value for mechanical complications should therefore be interpreted with caution. Second, the choice of instrumentation length was determined intraoperatively by the surgeon’s judgement rather than according to pre-defined, standardized criteria, introducing a potential selection bias that may have influenced outcomes; specifically, baseline differences between groups in age, fracture severity, neurological status, or ASD subtype—which were not formally compared—may have influenced both construct choice and observed outcomes, and this possibility should be borne in mind when interpreting the comparative results. Third, the small number of cemented cases (*n* = 3) precluded any meaningful statistical evaluation of the role of PMMA augmentation. Finally, the substantial loss to outpatient follow-up (52% of patients) may have led to an underestimation of late complications occurring beyond the 3-month assessment window.

## 5. Conclusions

In this single-center retrospective cohort, instrumentation length was associated with a statistically significant difference in mechanical complication rates in thoracolumbar ASD fractures treated with open posterior stabilization, although these findings should be interpreted with caution given the small sample size, marked group imbalance, and borderline *p*-value. The 3 + 3 configuration—with pedicle screw fixation at three levels above and three levels below the fracture—was associated with the lowest observed implant failure rate (4.35%) compared to the 2 + 2 configuration (22.22%). These results are consistent with the DGOU recommendation of a minimum of two fixation levels on each side of the fracture, and suggest that extending to three levels may confer an additional mechanical advantage; however, given the exploratory nature of this analysis, definitive superiority of the 3 + 3 construct cannot be established from the present data alone.

No significant association was identified between instrumentation length and infectious complications or 3-month mortality, indicating that these outcomes are predominantly driven by patient-related biological factors rather than by surgical construct design. The high mortality rate (29.7%) observed in this cohort underscores the clinical complexity and systemic vulnerability of ASD fracture patients and highlights the need for comprehensive perioperative multidisciplinary management.

Future research should priorities prospective, multicenter studies with larger and more balanced cohorts to definitively establish instrumentation length guidelines. Specific areas requiring further investigation include the following: (1) the role of intraoperative Hounsfield Unit-guided PMMA cement augmentation as a complement to extended fixation; (2) the potential for minimally invasive and navigation- or robot-assisted percutaneous instrumentation to preserve the mechanical advantages of the 3 + 3 configuration while reducing surgical morbidity; (3) the impact of systematic pharmacological optimization of bone quality (bisphosphonates, anti-osteoporotic agents) prior to elective stabilization; and (4) the development of ASD-specific scoring systems to guide individualized surgical decision-making. Only through such structured and collaborative research efforts will it be possible to optimize outcomes for this particularly challenging and growing patient population.

## Figures and Tables

**Table 1:** Main demographic and clinical characteristics of patients included in the study.

Characteristics	Patients (*n* = 37)
Sex (Male/Female)	28/9
Mean age (years)	77 ± 7.3
AO Fracture Type	B: 27 (11 B1/7 B2/10 B3); C: 10
Neurological impairment	7/37 (18.9%)
Mean follow-up (months)	30 ± 5.3
Instrumentation configuration	3 + 3: 23/3 + 2: 5/2 + 2: 9
Cement augmentation	3/37 (8.1%)
Mean instrumented segments	5.3 ± 0.5
Mean screw density	0.89 ± 0.06

## Data Availability

The data presented in this study are available on request from the corresponding author. The data are not publicly available due to privacy and ethical restrictions.

## References

[1] Rudwaleit M., Landewé R., Van Der Heijde D., Listing J., Brandt J., Braun J., Burgos-Vargas R., Collantes-Estevez E., Davis J., Dijkmans B. (2009). The development of Assessment of SpondyloArthritis international Society classification criteria for axial spondyloarthritis (part I): Classification of paper patients by expert opinion including uncertainty appraisal. Ann. Rheum. Dis..

[2] Feldtkeller E., Vosse D., Geusens P., Van Der Linden S. (2006). Prevalence and annual incidence of vertebral fractures in patients with ankylosing spondylitis. Rheumatol. Int..

[3] Muñoz-Ortego J., Vestergaard P., Rubio J.B., Wordsworth P., Judge A., Javaid M.K., Arden N.K., Cooper C., Díez-Pérez A., Prieto-Alhambra D. (2014). Ankylosing spondylitis is associated with an increased risk of vertebral and nonvertebral clinical fractures: A population-based cohort study. J. Bone Miner. Res..

[4] Caron T., Bransford R., Nguyen Q., Agel J., Chapman J., Bellabarba C. (2010). Spine fractures in patients with ankylosing spinal disorders. Spine.

[5] Teunissen F.R., Verbeek B.M., Cha T.D., Schwab J.H. (2017). Spinal cord injury after traumatic spine fracture in patients with ankylosing spinal disorders. J. Neurosurg. Spine.

[6] Rodrigues L.F., Moura-Neto V., e Spohr T.C.L.d.S. (2018). Biomarkers in Spinal Cord Injury: From Prognosis to Treatment. Mol. Neurobiol..

[7] Westerveld L., van Bemmel J., Dhert W., Oner F., Verlaan J. (2014). Clinical outcome after traumatic spinal fractures in patients with ankylosing spinal disorders compared with control patients. Spine J..

[8] Reinhold M., Knop C., Kneitz C., Disch A. (2018). Spine Fractures in Ankylosing Diseases: Recommendations of the Spine Section of the German Society for Orthopaedics and Trauma (DGOU). Glob. Spine J..

[9] Westerveld L.A., Verlaan J.J., Oner F.C. (2009). Spinal fractures in patients with ankylosing spinal disorders: A systematic review of the literature on treatment, neurological status and complications. Eur. Spine J..

[10] Vaccaro A.R., Oner C., Kepler C.K., Dvorak M., Schnake K., Bellabarba C., Reinhold M., Aarabi B., Kandziora F., Chapman J. (2013). AOSpine thoracolumbar spine injury classification system: Fracture description, neurological status, and key modifiers. Spine.

[11] Harms J., Melcher R.P. (2001). Posterior C1–C2 fusion with polyaxial screw and rod fixation. Spine.

[12] Lindtner R.A., Kammerlander C., Goetzen M., Keiler A., Malekzadeh D., Krappinger D., Schmid R. (2017). Fracture reduction by postoperative mobilisation for the treatment of hyperextension injuries of the thoracolumbar spine in patients with ankylosing spinal disorders. Arch. Orthop. Trauma Surg..

[13] Buxbaum R.E., Shani A., Mulla H., Rod A., Rahamimov N. (2021). Percutaneous, PMMA-augmented, pedicle screw instrumentation of thoracolumbar ankylotic spine fractures. J. Orthop. Surg. Res..

[14] Rosine N., Fogel O., Koturan S., Rogge L., Bianchi E., Miceli-Richard C. (2023). T cells in the pathogenesis of axial spondyloarthritis. Jt. Bone Spine.

[15] Barkay G., Apterman S., Ackshota N., Shtewe A.H., Sissman E., Friedlander A. (2023). Early surgery for thoracolumbar extension-type fractures in geriatric patients with ankylosing disorders reduces patient complications and mortality. Spine J..

[16] Ull C., Yilmaz E., Hoffmann M.F., Reinke C., Aach M., Schildhauer T.A., Kruppa C. (2022). Factors Associated with Major Complications and Mortality During Hospitalization in Patients with Ankylosing Spondylitis Undergoing Surgical Management for a Spine Fracture. Glob. Spine J..

[17] Chen S.R., Munsch M.A., Chen J., Couch B.K., Wawrose R.A., Oyekan A.A., Adjei J., Donaldson W.F., Lee J.Y., Shaw J.D. (2023). Spine Fractures of Patients with Ankylosing Spondylitis and Diffuse Idiopathic Skeletal Hyperostosis: Fracture Severity and Injury-Related Mortality at a Level I Trauma Center. Asian Spine J..

[18] Sharma M., Jain N., Wang D., Ugiliweneza B., Boakye M., Drazin D. (2022). Impact of age on mortality and complications in patients with Ankylosing Spondylitis spine fractures. J. Clin. Neurosci..

[19] Klingberg E., Nurkkala M., Carlsten H., Forsblad-D’Elia H. (2014). Biomarkers of bone metabolism in ankylosing spondylitis in relation to osteoproliferation and osteoporosis. J. Rheumatol..

[20] Quevedo Mayorga P.A., González A.A., Mora Méndez J.M., Quevedo J.C.V., Moya L.M.E., Quintero A.M., Morón C.E.G., Moreno C.A.C. (2026). Diagnosis of osteoporosis in patients with ankylosing spondylitis: Systematic review, and meta-analysis. Rev. Colomb. Reumatol..

[21] Svac J., Stranak P., Hrin T., Hrabalek L., Alberty R., Zamborsky R., Kilian M. (2024). The effect of lengthening of the percutaneous implant in the surgical treatment of Th-L ankylosed spine fractures: 4 segment fixation versus 5 to 8 segment fixation. Clin. Study.

[22] Sulpis B., Neri T., Klasan A., Castel X., Vassal F., Tetard M.C. (2024). Isolated posterior stabilization in type B and C thoracolumbar fractures associated with ankylosing spine disorders: A single center experience with clinical and radiological outcomes. SICOT-J.

[23] Daher M., Rezk A., Baroudi M., Gregorczyk J., Criss M.B., McDermott J., Mcdonald C.L., Diebo B.G., Daniels A.H. (2024). Management of Thoracolumbar Vertebral Fractures and Dislocations in Patients with Ankylosing Conditions of the Spine. Orthop. Rev..

[24] Hasegawa J., Suzuki M., Kishimoto K., Sato R., Ohno Y., Sugiura T., Yamamoto H., Terabe K., Asai S., Imagama S. (2025). Evaluation of Vertebrae in Patients with Ankylosing Spondylitis Using Hounsfield Unit Values. Int. J. Rheum. Dis..

[25] Coniglio A., Rava A., Fusini F., Colò G., Massè A., Girardo M. (2021). Effectiveness and reliability of cannulated fenestrated screws augmented with polymethylmethacrylate cement in the surgical treatment of osteoporotic vertebral fractures. J. Craniovertebr. Junction Spine.

